# Supplementary figures and images for: A 3D human iPSC-derived multi-cell type neurosphere system to model cellular responses to chronic amyloidosis
Source: J Neuroinflammation. 2025 Apr 24;22:119. doi: 10.1186/s12974-025-03433-3 (PMC12023538; doi:10.1186/s12974-025-03433-3)

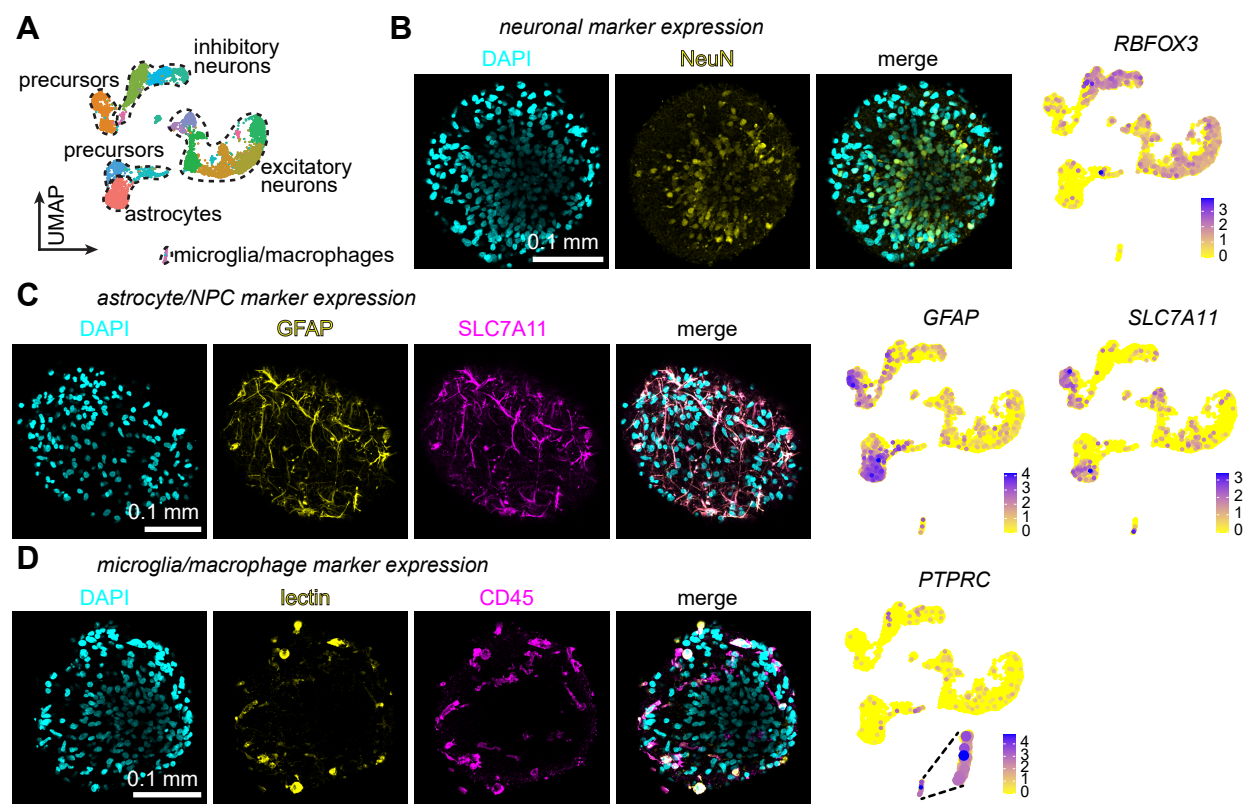

Supplement: Supplementary file 1 — Supplementary Material 1: Figure S1: Alternative marker expression patterns confirming the presence of neurons, astrocytes and microglia-like cells in hiNS. A: Cell population annotations from our snRNA-seq data set. B: Neuronal nuclei labeled by immunostaining for NeuN and its gene expression pattern shown on the right. C: Astrocyte/NPC expression of SLC7 A11 overlaps with GFAP labelled by immunostaining and their gene expression patterns shown on the right. D: Microglia-like cells were labelled with lectin, overlapping with CD45 immunostaining and its gene expression pattern shown on the right [file 12974_2025_3433_MOESM1_ESM.pdf]

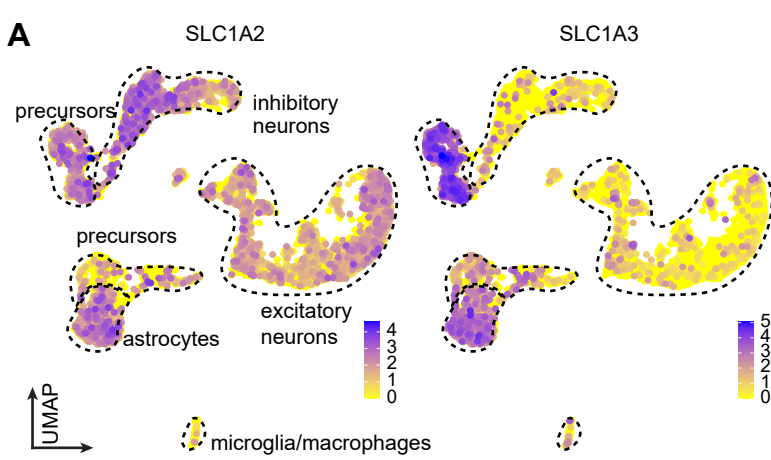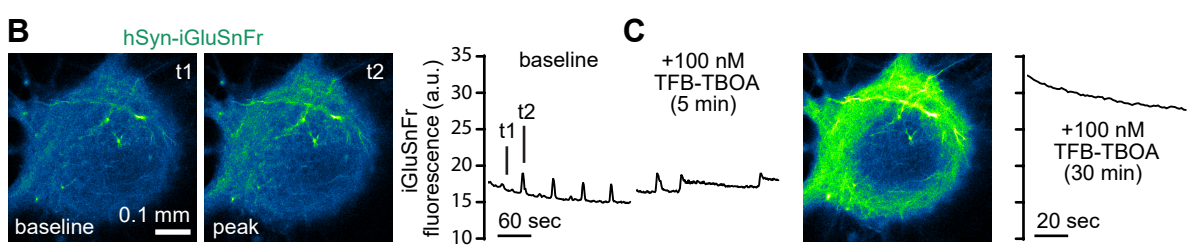

Supplement: Supplementary file 2 — Supplementary Material 2: Figure S2: Maintaining neural wave activity requires glutamate transporter activity in hiNS. A: Glutamate transporter EAAT2and EAAT1expression in hiNS. While SLC1 A2 expression is widespread in neurons, progenitors and astrocytes, SLC1 A3 expression appears more specific for progenitor and astrocyte cell populations. B: iGluSnFr fluorescence, indicating extracellular glutamate waves. C: Extracellular glutamate levels gradually increase by blocking EAAT1/2 with 100 nM TFB-TBOAwhich prevents wave formation within minutes in hiNS indicating a functional role of glutamate transporters in maintaining calcium/glutamate wave activity [file 12974_2025_3433_MOESM2_ESM.pdf]

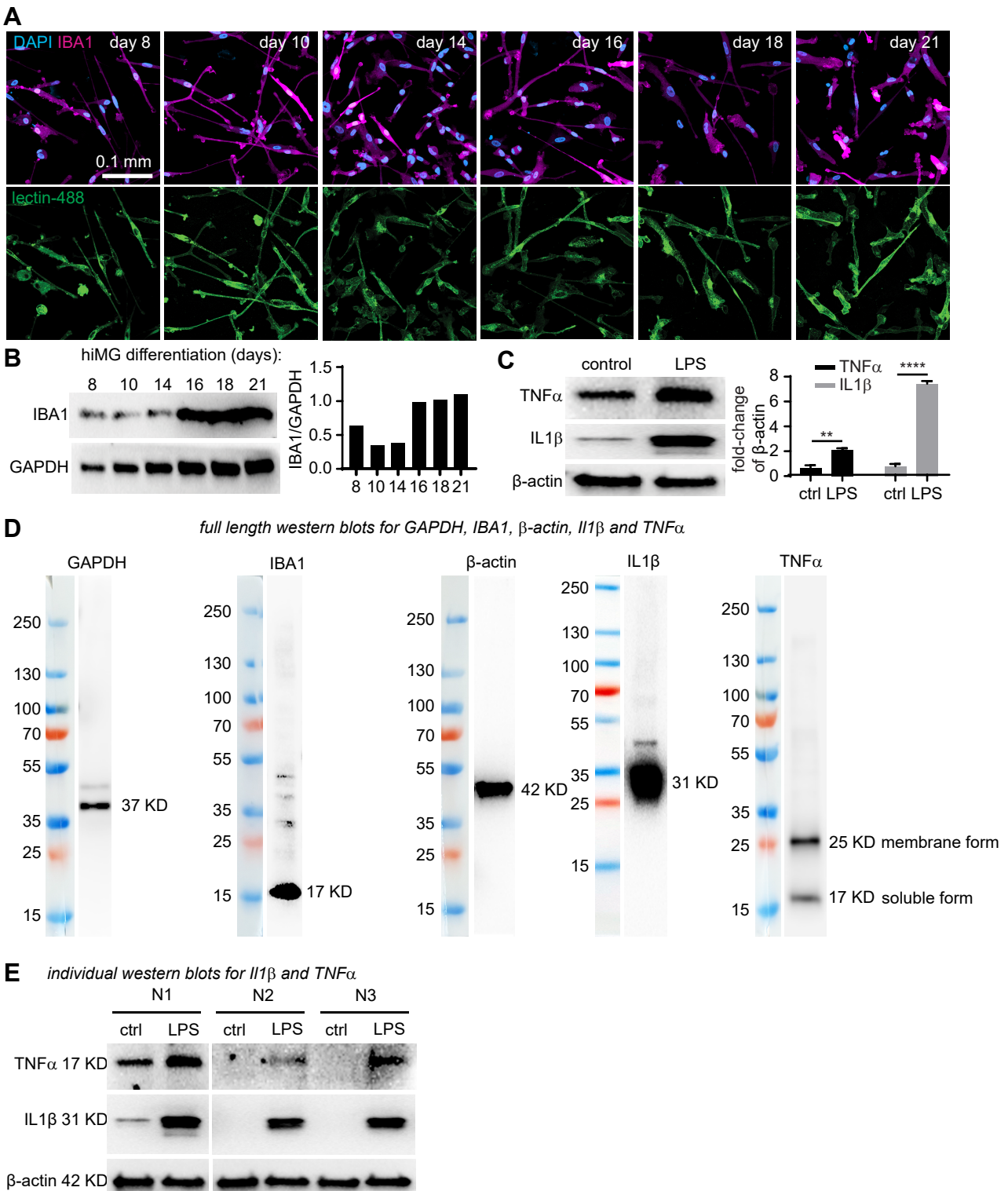

Supplement: Supplementary file 3 — Supplementary Material 3: Figure S3: hiMG differentiation timeline shows a gradual increase in IBA1 expression. A: hiMG were plated on coverslips and fixed at different time points during differentiation. Immunofluorescence for IBA1is detectable at all time points. Co-staining with tomato lectin- 488 confirms that lectin can be used to label hiMG in vitro. B: Western blot for IBA1 confirms stable expression in hiMG up to at least day 21. C: Stimulation of hiMG with LPS for 24 h results in significant TNFα and IL1β release confirming their capacity to react to proinflammatory stimuli. D: Full length blots for GAPDH, IBA1, β-actin, Il1β and TNFα. E: All individual blots for Il1β and TNFα used for the quantification in panel C [file 12974_2025_3433_MOESM3_ESM.pdf]

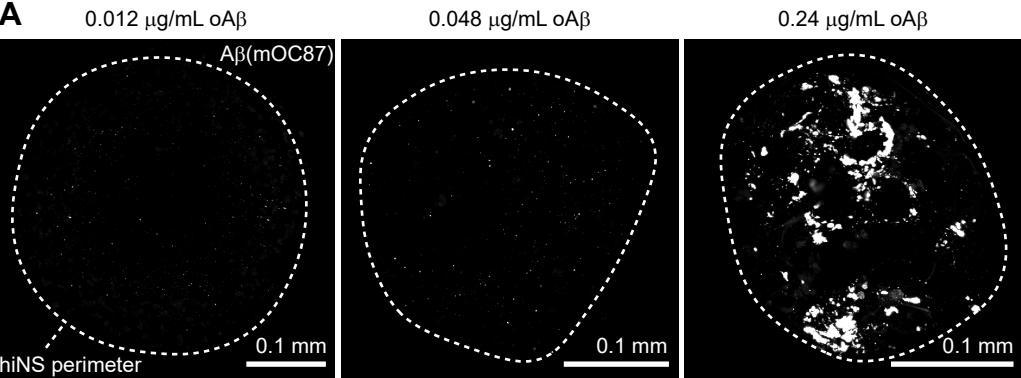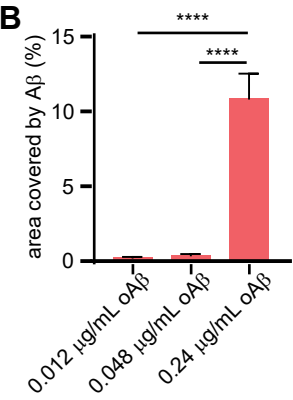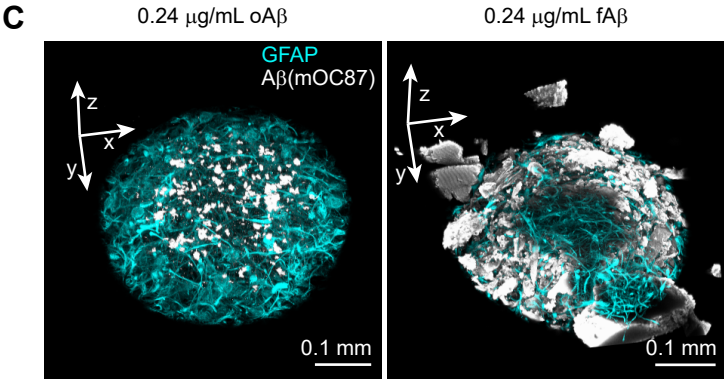

Supplement: Supplementary file 4 — Supplementary Material 4: Figure S4: Aβ aggregates form within 7 days of oligomeric Abtreatment in hiNS. A: hiNS cell culture medium was supplemented with three different doses of oAβ. Only the highestconcentration resulted in robust formation of aggregated Aβpositive area in hiNS. One-way ANOVA followed by Holm-Sidak’s post-hoc test C: Three-dimensional reconstruction of Aβ aggregates in hiNS. While oAβ treatment results in plaque-like aggregates inside the tissue, supplementing cell culture media with pre-aggregated fibrillar Aβresults in large Aβ sheets covering the hiNS tissue which do not mimic plaque-like structures [file 12974_2025_3433_MOESM4_ESM.pdf]

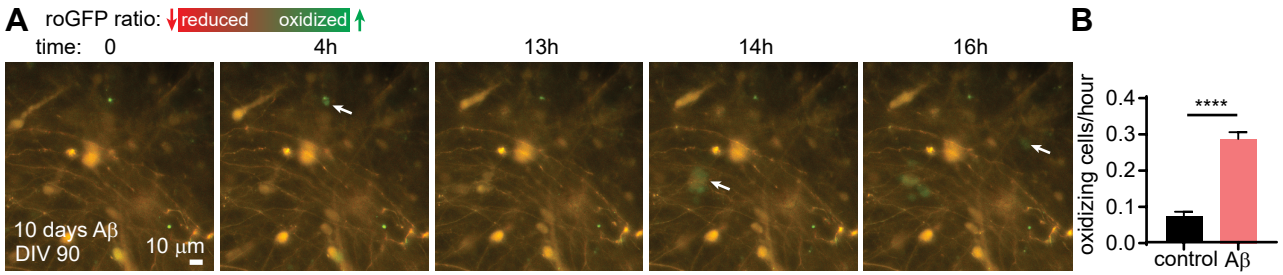

Supplement: Supplementary file 5 — Supplementary Material 5: Figure S5: Over-night live confocal imaging of hSyn -roGFP1 expressing hiNSduring chronic Aβ treatment. Recordings of 18 - 22 h duration were conducted between DIV 87 - 104 with chronic Aβ treatment ranging from 7 to 23 days. A: Example section of an Aβ treated hiNS displaying multiple neurons oxidizing during the recording time period. B: The total number of oxidizing cells per hour was quantified for both Aβ treated as well as control hiNS. In Aβ treated spheres 0.29 ± 0.02 cells oxidized per hour compared to 0.07 ± 0.01 under control conditions [file 12974_2025_3433_MOESM5_ESM.pdf]

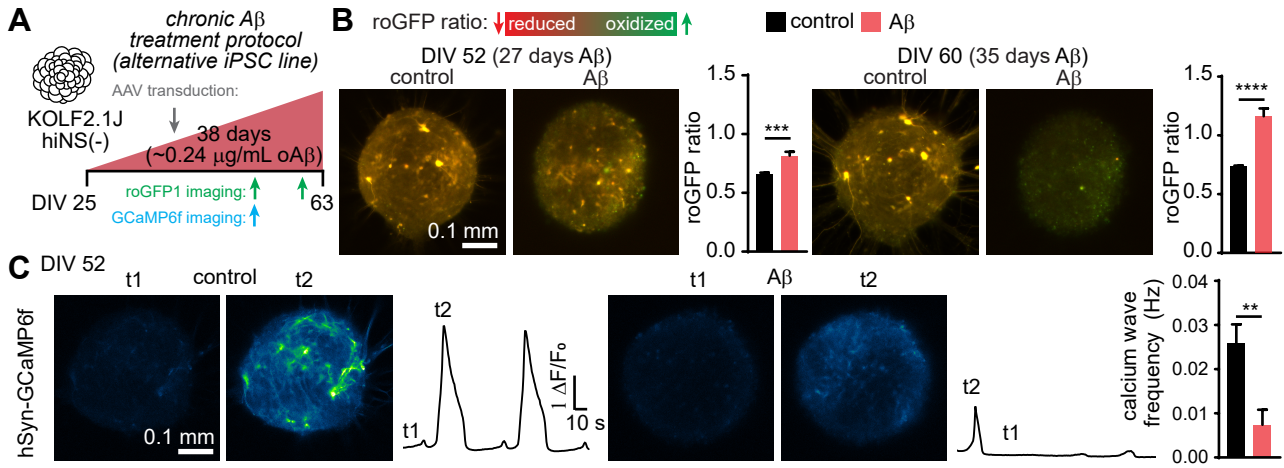

Supplement: Supplementary file 6 — Supplementary Material 6: Figure S6: Chronic amyloidosis effects on hiNS derived from an alternative iPSC line). A: We employed our 5w Aβ treatment strategy and roGFP1 and GCaMP6f imaging to monitor Aβ induced neurodegeneration. B: Significant manifestation of oxidative stress, evident by an increase in roGFP ratios, after 27 days of Aβ exposure. Continuous Aβ exposure results in progressively more oxidative stress after 35 days of treatment. C: KOLF2.1 J hiNSdisplay calcium wave activity which frequencies get significantly reduced by chronic Aβ treatment. Statistical testing was performed using an unpaired student’s t-tests [file 12974_2025_3433_MOESM6_ESM.pdf]

ctrl hiNS(-)

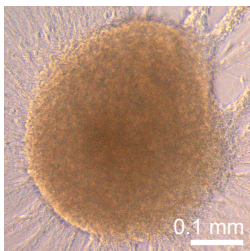

3w A $\beta$  hiNS(-)

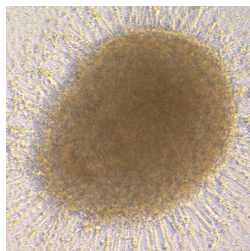

5w A $\beta$  hiNS(-)

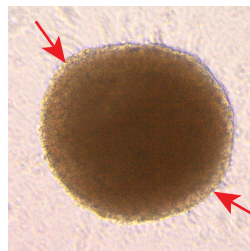

ctrl hiNS(+)

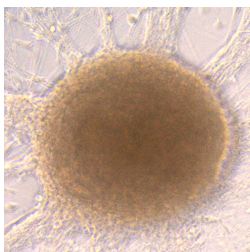

3w A $\beta$  hiNS(+)

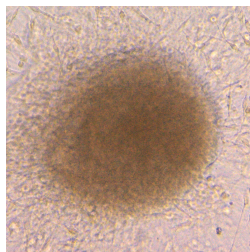

5w A $\beta$  hiNS(+)

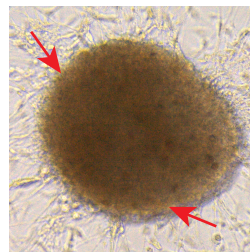

Supplement: Supplementary file 7 — Supplementary Material 7: Figure S7: Chronic amyloidosis does not result in Aβ induced Tau hyperphosphorylation. A: Immunofluorescence staining for phosphorylated Tau T181and total Tau in hiNS of all 6 experimental conditions. B: Quantification of pTau181/total Tau ratio in hiNS indicates pTau modulation by hiMG independent of Aβ exposure. C: Soluble pTau181 was quantified using ELISA on supernatants from hiNSconfirms the lack of Aβ induced Tau hyperphosphorylation [file 12974_2025_3433_MOESM7_ESM.pdf]

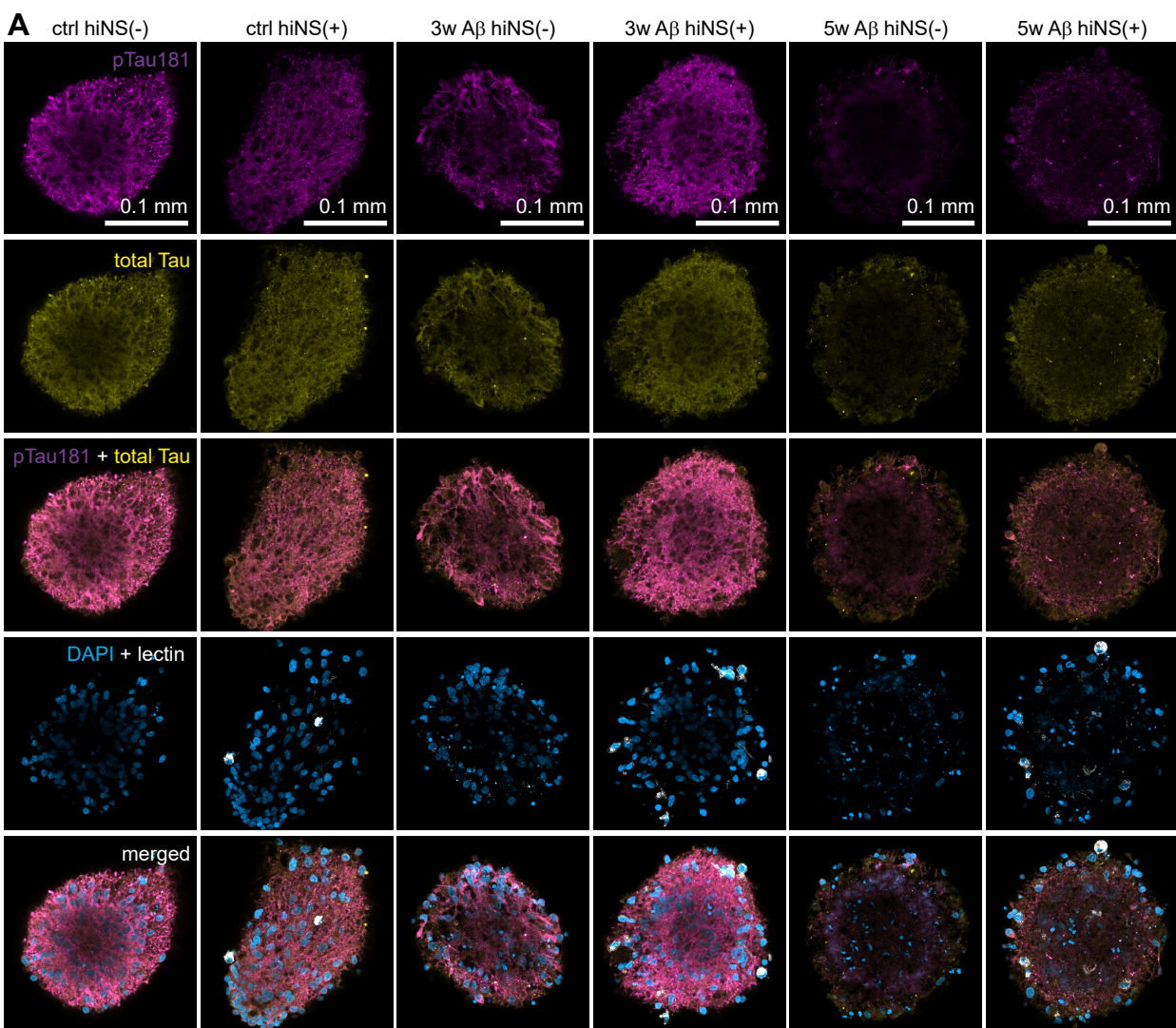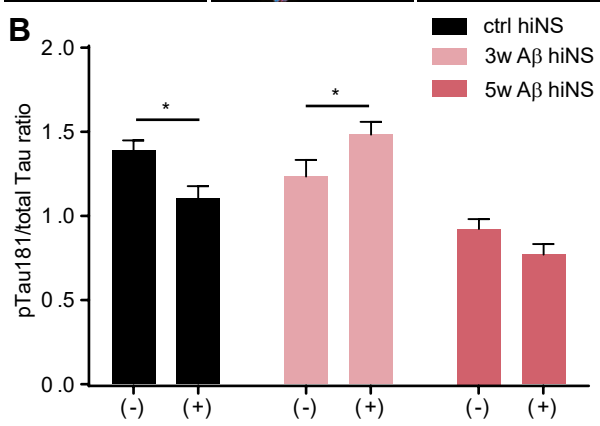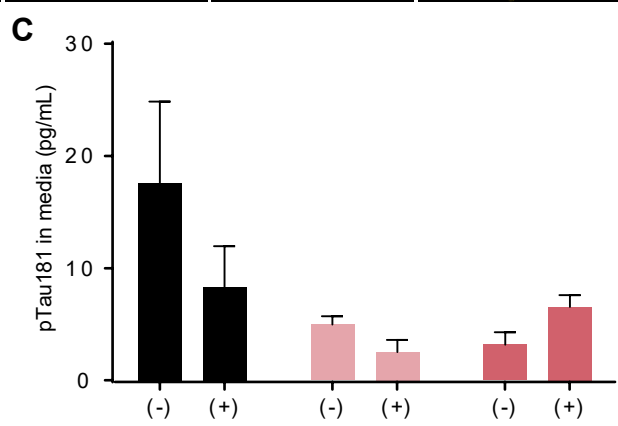

Supplement: Supplementary file 8 — Supplementary Material 8: Figure S8: Example transmitted light images of control and Aβ treated hiNS with and without hiMG. hiMG attach to the surface surrounding hiNS Note that 5w Aβ hiNSdisplay a darker contrast and appear to detach from the surface [file 12974_2025_3433_MOESM8_ESM.pdf]

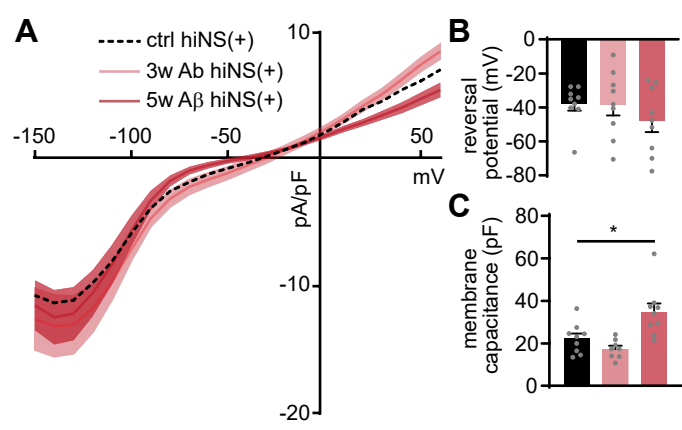

Supplement: Supplementary file 9 — Supplementary Material 9: Figure S9: Electrophysiological properties of hiMG in 3w and 5w Aβ hiNS. A: Current-voltage relationship of hiMG ranging from - 150 to + 50 mV after chronic Aβ treatment appears similar to hiMG from ctrl hiNSwith no significant increase in outward or inward currents. B: Reversal potentials of hiMG do not differ between treatment groups indicating membrane potentials of ~- 40 mV. c: Membrane capacitance of hiMG was significantly increased in hiMG of 5w Aβ hiNSindicating potentially larger cell sizes with prolonged Aβ treatment: N= 9 cells each) [file 12974_2025_3433_MOESM9_ESM.pdf]

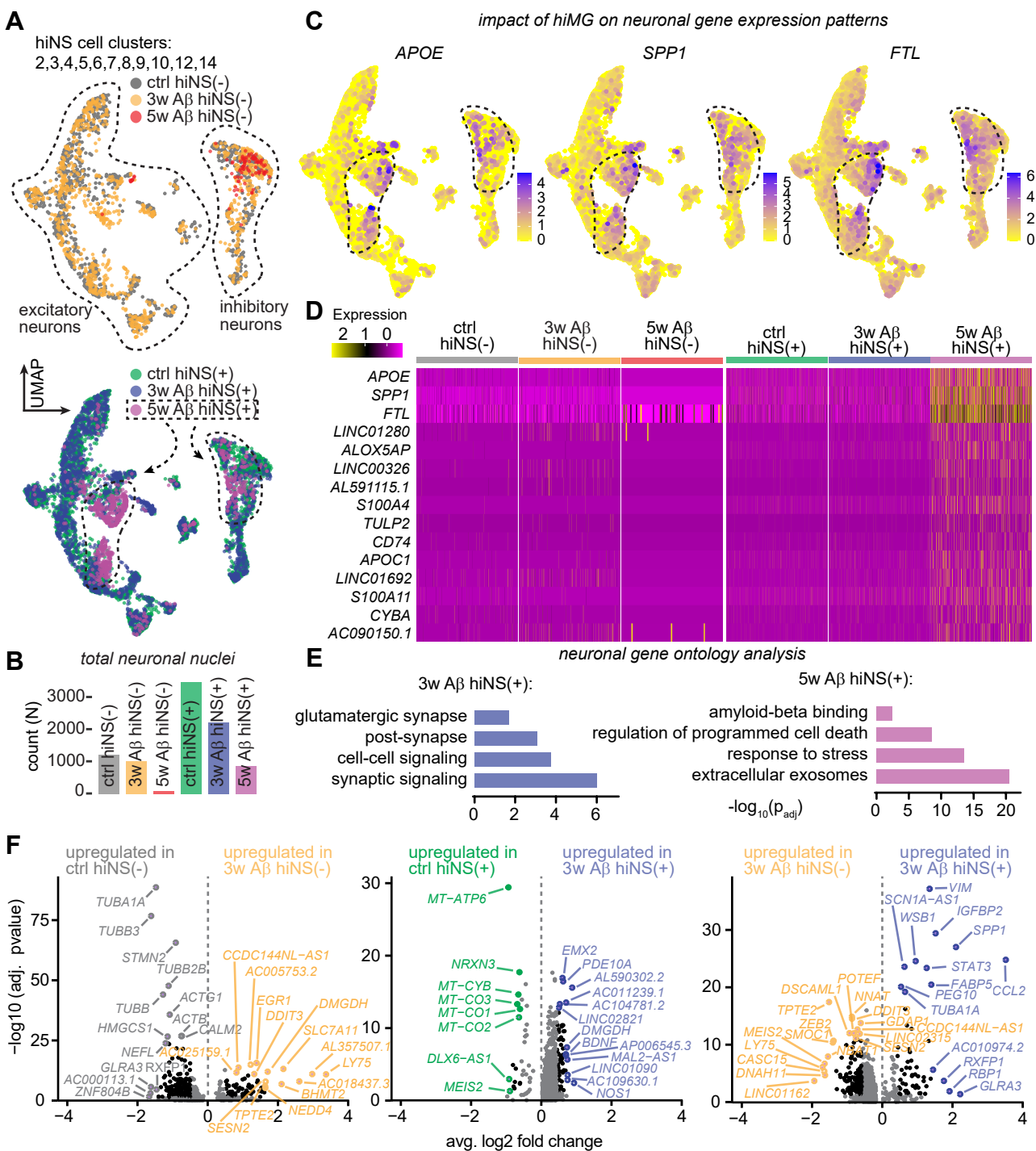

Supplement: Supplementary file 10 — Supplementary Material 10: Figure S10: Neuronal gene expression changes during chronic amyloidosis in the presence of hiMG include APOE. A: Isolated neuronal cell populations from snRNA-seq on hiNSin ctrl, 3w and 5w Aβ conditions. Merged UMAPs of all 6 experimental groups displaying excitatory neuronsand inhibitory neurons. Note that the majority of 5w Aβ hiNSneurons cluster together in the individual neuronal populations. B: Quantification of total neuronal nuclei count in all 6 experimental groups. Note that only few neurons remain after chronic amyloidosis in the absence of hiMG. C: UMAP overlay of the three most significant DEGs APOE, SPP1 and FTL. Cell populations enriched with 5w Aβ hiNSneurons are encircled in dashed black lines. D: Heatmap depicting the top 15 most significant DEGs in 5w Aβ hiNSneurons, with APOE being the most significant upregulated gene. E: Neuronal gene ontology analysis in 3w/5w Aβ hiNSneurons. F: Direct comparison of neuronal DEGs depending on Ab or hiMG for ctrl and 3w Aβ hiNS shown in volcano plots. Top 10 DEGs in respect to fold change and/or adjusted pvalue are labeled. Note that AD-associated genes, such as CCL2, STAT3 and VIM, are only upregulated if hiMG were present during Aβ treatment [file 12974_2025_3433_MOESM10_ESM.pdf]

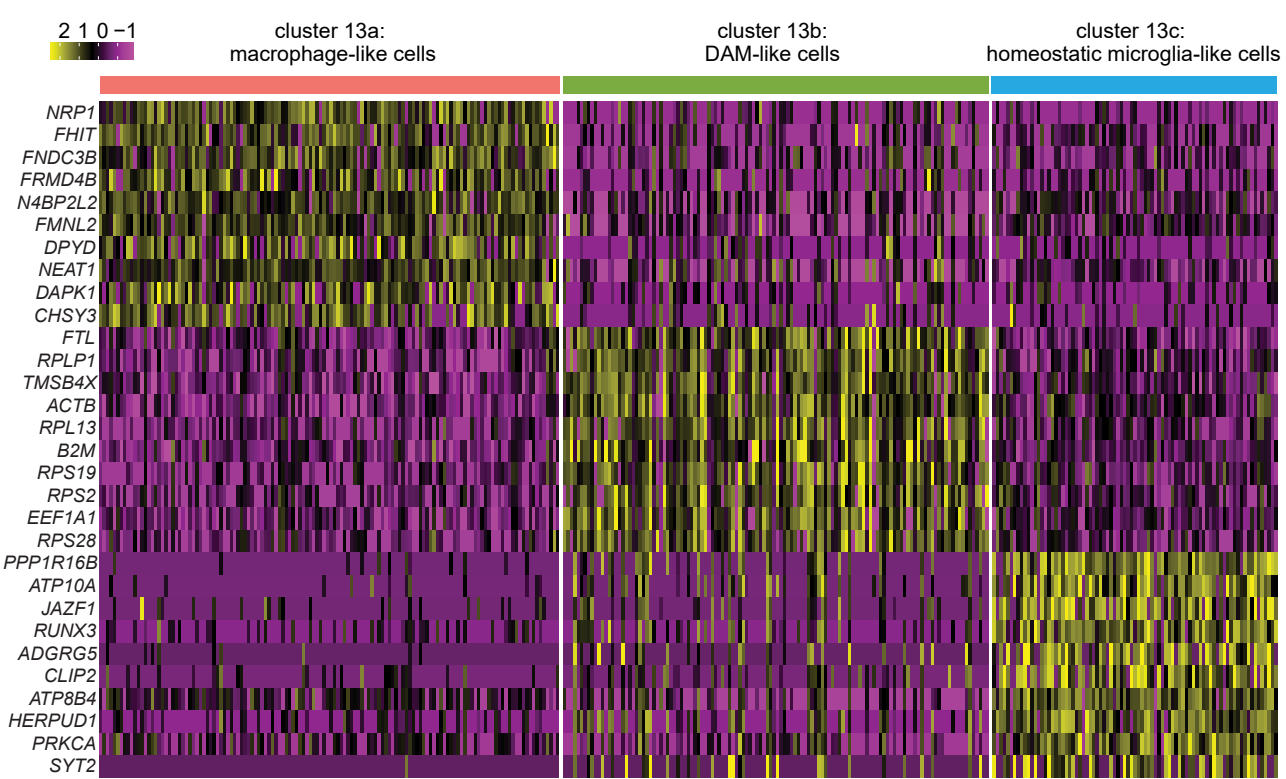

Supplement: Supplementary file 11 — Supplementary Material 11: Figure S11: Top 10 most significant DEGs per subcluster of hiMG from 5w Aβ hiNSdisplayed in a heatmap [file 12974_2025_3433_MOESM11_ESM.pdf]

**A** full length western blot  
for APOE

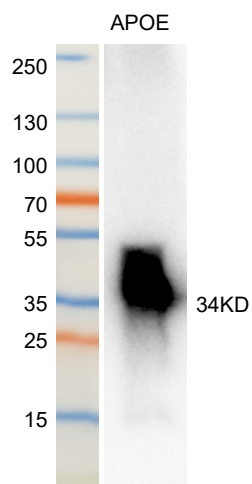

**B** all individual western blots for APOE

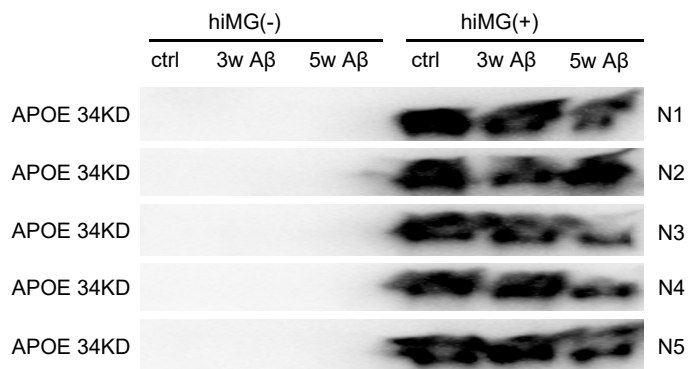

Supplement: Supplementary file 12 — Supplementary Material 12: Figure S12: Additional western blots from hiNS supernatant for APOE. A: Full length blot for APOE. B: All individual blots used for the soluble APOE quantification as shown in Fig. 8C. [file 12974_2025_3433_MOESM12_ESM.pdf]
